# Acidic Microenvironment Regulates the Severity of Hepatic Ischemia/Reperfusion Injury by Modulating the Generation and Function of Tregs via the PI3K-mTOR Pathway

**DOI:** 10.3389/fimmu.2019.02945

**Published:** 2020-01-09

**Authors:** Xiaojie Gan, Rongsheng Zhang, Jian Gu, Zheng Ju, Xiao Wu, Qi Wang, Hao Peng, Jiannan Qiu, Jinren Zhou, Feng Cheng, Ling Lu

**Affiliations:** ^1^Hepatobiliary Center, The First Affiliated Hospital of Nanjing Medical University, Nanjing, China; ^2^Research Unit of Liver Transplantation and Transplant Immunology, Chinese Academy of Medical Sciences, Nanjing, China; ^3^Key Laboratory of Liver Transplantation, Chinese Academy of Medical Sciences, Nanjing, China; ^4^Key Laboratory of Living Donor Liver Transplantation, National Health Commission (NHC), Nanjing, China

**Keywords:** acidic microenvironment, Treg, hepatic ischemia/reperfusion injury, immune cells, signaling

## Abstract

Hepatic ischemia/reperfusion injury (HIRI) is a major cause of liver dysfunction and even liver failure after liver transplantation and hepatectomy. One of the critical mechanisms that lead to HIRI is an acidic microenvironment, which develops due to the accumulation of high acid-like substances such as lactic acid and ketone bodies. Previous studies have shown that the adoptive transfer of induced regulatory T cells (iTregs) attenuates HIRI; however, little is known about the function of Tregs in the acidic microenvironment of a HIRI model. In the present study, we examined the effect of acidic microenvironment on Tregs *in vitro* and *in vivo*. Here, we report that microenvironment acidification and dysfunction of the liver is induced during HIRI in humans and mice and that an acidic microenvironment can inhibit the generation and function of CD4^+^CD25^+^Foxp3^+^ iTregs via the PI3K/Akt/mTOR signaling pathway. By contrast, the reversal of the acidic microenvironment restored Foxp3 expression and iTreg function. In addition, the results of cell culture *in vitro* indicated that the proton pump inhibitor omeprazole improves decreased iTreg differentiation caused by the acidic microenvironment, suggesting the potential clinical use of proton pump inhibitors as immunoregulatory therapy in the treatment of HIRI. Furthermore, our findings demonstrate that buffering the acidic microenvironment to attenuate HIRI in mice has an inseparable relationship with Tregs. Thus, an acidic microenvironment is a key regulator in HIRI, involved in modulating the generation and function of Tregs.

## Introduction

Hepatic ischemia/reperfusion injury (HIRI) refers to a clinical phenomenon wherein tissue damage is aggravated and even irreversible after the liver restores blood flow on the basis of ischemia. HIRI is an inevitable pathophysiological process during liver resection and liver transplantation, which directly affects the prognosis of surgery. The mechanism of HIRI is complex, especially in immune cells. In the early stage of HIRI (within 2 h after reperfusion), Kupffer cells synthesize reactive oxygen species (ROS) and pro-inflammatory factors such as TNF-α and IL-1 via the TLR4/NF-κB pathways to activate hepatic sinusoidal endothelial cells (1, 2). Subsequently, the expression of intercellular adhesion molecule 1 and vascular cell adhesion molecule one increases, causing the local accumulation of neutrophils in the liver and the release of myeloperoxidase (such as Cl^−^). In turn, these actions catalyze the formation of strong oxidants such as HOCl by H_2_O_2_, aggravating endothelial cells injury and eventually leading to persistent ischemic injury to the liver (3). In addition, the direct induction of master cell degranulation by ROS may also contribute to the progress of HIRI (4). Recent studies have found that T cells are probably the primary mediator of HIRI (5) and that the reduction or elimination of CD4^+^ T cells can effectively reduce neutrophil aggregation, inhibit neutrophil activation, and improve HIRI (6). Furthermore, Kun et al. found that exosomes produced by hUCB-MSCs alleviated HIRI via upregulating regulatory T cell (Treg) differentiation and inhibiting Th17 cell differentiation (7).

Tregs are a subset of T cells that maintain the balance of the body's immune system. Changes in the ratio and function of Tregs can cause many diseases such as autoimmune diseases. They usually can be divided into two classes: natural Tregs and inducible Tregs (iTregs). Natural Tregs are derived from the thymus, also known as thymic Tregs. iTregs, also recognized as peripheral Tregs *in vivo*, are formed by the development and differentiation from naïve T cells in the peripheral environment within TGF-β (8). Both classes have the same CD4^+^CD25^+^CD127^low^ phenotype and express the transcription factor fork head box P3 (Foxp3), a characteristic marker. Various studies *in vivo* or *in vitro* have shown that both classes can exert immunosuppressive effects by inhibiting the activation of immune cells such as T cells, B cells, natural killer cells, and dendritic cells (9). Furthermore, recent studies have demonstrated that the adoptive transfer of iTregs can alleviate HIRI in mice (10). Due to the shortage of thymic Tregs and the similar effect of iTregs, we focused on iTregs in our research.

In mammals, the pH values of blood and tissue are usually maintained within a narrow range around 7.4. However, some diseased areas, such as inflammation loci, cancer nests, and infarct areas, have shown to be acidified. The pH values of the interstitial fluid of tumors and abscesses can decrease lower than 6.0, averaging 0.2–0.6 units less than the mean extracellular pH of normal tissues (11). In various cancers, acidification of the tumor microenvironment stimulates tumor cell aggression and malignant progression through remodeling of the extracellular matrix (ECM), promotion of angiogenesis, and dedifferentiation of cancer cells, resulting in increased invasiveness and metastasis (12). A recent study on lung cancer reported that prolonged exposure to an acidic environment induces a sustained invasive phenotype through a mechanism differing from that of reversible phenotype resulting from transient exposure to acidic extracellular pH (13). In an *in vitro* study, the growth of bone marrow stem cells from a patient with pancytopenia was found to be inhibited by concentrations of methylmalonic acid found *in vivo* (14). In addition, impaired neutrophil and monocyte chemotaxis has been found in some patients with methylmalonic aciduria (15). Furthermore, Menkin demonstrated that the number of granulocytes in local exudates decreased as the local pH decreased and found higher-than-normal concentrations of lactic acid and hydrogen ion in exudates from patients with diabetes (16).

Given that the acidic microenvironment is one of the key mechanisms leading to HIRI, and the intriguing, but not yet fully understood, relationship between Treg and acidic microenvironment in HIRI, we designed a series of experiments *in vitro* and *in vivo* to investigate the role of acidic microenvironment in HIRI. Overall, we found that an acidic microenvironment is a key regulator in HIRI, involved in modulating the generation and function of Tregs. Importantly, the buffering of the acidic microenvironment to attenuate HIRI in mice was associated with restoration of Tregs function.

## Materials and Methods

### Animals and Liver Partial Ischemia/Reperfusion Injury Model

Male mice (C57BL/6), 8 weeks old, were purchased from the Model Animal Research Center of Nanjing University. The mouse model of partial HIRI was established as follows (17). Mice were anesthetized with 3.5% chloral hydrate (0.1 ml/10 g) and then the midline incision was performed. The middle and left lateral portal vein and hepatic artery were clamped by the non-invasive vascular clip for 60 min to induce 70% of the liver ischemia. After ischemia, reperfusion was initiated by removing the clip. In addition to vascular clamping, the same protocol was used in sham mice. For NaHCO_3_ treatment and NaHCO_3_ + PC61 treatment groups, mice were injected with 5% NaHCO_3_ solution (100 μl/10 g, pH 8.2, Sigma) through caudal vein at the beginning of ischemia with or without PC61 (250 mg/mouse, intraperitoneal injection, Sigma) pretreatment for 3 days. Mice ischemic liver tissue and blood were collected at the indicated time. The sample size (*n*) for each experimental group is 6. Investigators were blinded to the group allocation when assessing the outcome. All animals were treated according to the guidelines for the use of experimental animals and approved by the Institutional Animal Care and Research Advisory Committee of Nanjing Medical University.

### Patient Recruitment and pH Measurement

Twenty patients who underwent partial hepatectomy were recruited from the Hepatobiliary Center of the First Affiliated Hospital of Nanjing Medical University. These patients were randomly grouped by hepatic portal occlusion within 20 min or more than 40 min. Informed consent was signed before surgery and approved by the Ethics Committee of the First Affiliated Hospital of Nanjing Medical University. Once the liver tissue was excised, the pH measurement was performed using a pH meter (pH5S, SanXin) immediately.

### Serum Transaminase

The mouse blood was harvested via eyeballs at the indicated time after reperfusion. After standing for 2 h at room temperature, the serum was extracted by centrifugation at 7,000 rpm for 10 min at 4°C. The serum alanine aminotransferase (ALT) and aspartate aminotransferase (AST) were detected by an automatic biochemical analyzer to evaluate the degree of liver injury.

### Naïve T Cells Isolation and Induction

Peripheral blood mononuclear cells (PBMCs) were prepared from the heparinized venous blood of healthy adult volunteers by Ficoll-Hypaque (Amersham Biosciences) density gradient centrifugation. Human CD4^+^CD45RA^+^ naïve T cells were sorted from PBMCs by using the CD4^+^ T cell isolation kit (Miltenyi) and CD45RA microbeads (Miltenyi) through auto-MACS (Miltenyi Biotec, Germany) in a two-step procedure of magnetic beads sorting (purity >95%). Naïve T cells were activated and induced to iTregs with anti-CD3/CD28 mAb-coated Dynabeads (1 bead to 5 cells, Gibco) in the presence of recombinant IL-2 (100 IU/ml, R&D) and TGF-β (1 ng/ml, R&D) in lymphocyte serum-free medium (Corning) with 10% fetal bovine serum (Sigma) for 72 h.

### Cells Culture Under Different pH Conditions

Media for cell culture at diverse pH values were prepared as follows. To minimize the pH change degree during the cell culture, 10 mM HEPES [4-(2-hydroxyethyl)-1-piperazine ethanesulfonic acid] was added to lymphocyte serum-free medium (pH 7.5 ± 0.2, Corning). After the addition of fetal bovine serum to media, HCl and NaOH were used to adjust the pH value from 6.5 to 8.5 using a pH meter. Naïve T cells were divided into six groups when treated with varying concentrations of omeprazole (Merck & Millipore): pH 7.5 + 10 μmol/L, pH 6.5 + 0 μmol/L, pH 6.5 + 10 μmol/L, pH 6.5 + 20 μmol/L, pH 6.5 + 40 μmol/L, and pH 6.5 + 80 μmol/L. In all experiments, the pH 6.5/7.5 group represents cells first cultured in a preconditioned medium (pH 6.5) and then were returned to approximate physiological pH (7.5) for 24 h before the assay. Samples analyzed per cell group, *n* = 8. Cells were cultured in 48-well plates (total volume 500 μl per well) at a density of 1 million/ml in the presence of 5% CO_2_ at 37°C.

### *In vitro* Suppression Assay

Human naïve CD4^+^ T cells sorted from healthy adult volunteers' PBMCs by auto-MACS (Miltenyi Biotec, Germany) were labeled with carboxyfluorescein diacetate succinimidyl ester (CFSE) (Invitrogen) and cultured in a 48-well bottom plate with anti-CD3/CD28-conjugated beads at a cell-to-bead ratio of 1:1. Serially diluted iTregs were co-cultured for 72 h, and cellular proliferation by CFSE was examined through flow cytometry.

### Phospho-Specific Protein Microarray

We used a phospho-specific protein microarray (PEX100, Full Moon BioSystems, USA) containing 1,318 antibodies against 584 phosphorylation sites of 432 proteins for phosphorylation profiling. The antibody array experiment was performed by Wayen Biotechnology (Shanghai, China) according to an established protocol. The SureScan Dx Microarray Scanner was used to scan for chip images, and the raw data were acquired using GenePix Pro v6.0 software. The phosphorylation ratio of each protein was calculated as follows: phosphorylation ratio = phospho value/unphospho value.

### Flow Cytometry

For extracellular staining, harvested cells were washed and incubated in PBS containing 1% FBS containing the below fluorochrome-conjugated antibodies in a flow tube. For intracellular staining of cytokines, cells were stimulated with phorbol 12-myristate 13-acetate (50 ng/ml, Biogems), ionomycin calcium salt (1 μg/ml, Biogems), and brefeldin A (5 μg/ml, Biogems) for 6 h. Then, cells were stained with surface markers and further fixed/permeabilized (BioLegend) and stained for intracellular protein. Human-specific monoclonal antibodies used for flow cytometry included CD4 (PE/cy7), CD25 (APC/cy7), Foxp3 (AF647), IL-10 (PE), TGF-β1 (AF488), CD45RA (APC), Annexin V (Pacific blue), propidium iodide (PI) purchased from BioLegend, and CD127 (FITC) purchased from BD Pharmingen. Sample detection was performed by MACSQuant Analyzer 10 (Miltenyi Biotec, Germany) and data were analyzed with FlowJo software.

### Quantitative Real-Time PCR

RNA was extracted from harvested cells using EasyPure RNA Kit (Transgen Biotech) according to the manufacturer's instructions. cDNA was synthesized using TransScript First-Strand cDNA Synthesis SuperMix (Transgen Biotech) and amplified by qRT-PCR in an ABI prism system (Applied Biosystems, Foster City, CA). Data were analyzed using the relative gene expression method and were normalized with GAPDH Ct values in the samples. The measurements of each sample were performed in triplicate. Primer sequences used for PCR amplification are as follows:

Foxp3, 5′-TCACCTACGCCACGCTCAT-3′
and 5′-AAGGCAAACATGCGTGTGAA-3′;
Bcl2, 5′-GGGAGGATTGTGGCCTTCTT-3′
and 5′-TCATCCACAGGGCGATGTT-3′;
Mcl1, 5′-GAGTTCTTCCATGTAGAGGACCTAGAA-3′
and 5′-TTATTAGATATGCCAAACCAGCTCCTA-3′;
Bax, 5′-TGTTTTCTGACGGCAACTTCA-3′
and 5′-CAGTTCCGGCACCTTGGT-3′;
Bad, 5′-CGAGTTTGTGGACTCCTTTAAGAAG-3′
and 5′-TCCCACCAGGACTGGAAGAC-3′;
Gapdh, 5′-CCATCTTCCAGGAGCGAGATC-3′;
and 5′-GCCTTCTCCATGGTGGTGAA-3′.

### Western Blot Analysis

Protein was extracted from harvested cells and their concentration was determined by the BCA assay (Pierce). Protein samples (30 μg) were resolved by SDS-PAGE and transferred to a PVDF membrane. The following antibodies were used: PI3K p85 (Cell Signaling Technology, CST#4257), P-PI3K p85/p55 (Tyr467/199) (Arigo, ARG66216), P-Akt (Ser473) (CST#4060), Total mTOR (CST#2983), P-mTOR (Ser2448) (CST#5536), P-mTOR (Thr2446) (Merck&Millipore, 09-345), P-mTOR (Ser2481) (CST#2974), P-p70S6K (Thr389) (CST#9234), P-4E-BP1 (Thr37/46) (CST#2855), ATP6V1H (v-ATPaseH) (Proteintech, 26683-1-AP), and GAPDH (CST#5174). HRP-conjugated goat anti-rabbit IgG (CST#7074) was used as the secondary antibody.

### Histopathology, Immunohistochemical Staining, and Analysis

Liver tissue collected from ischemia/reperfusion injury in mice was fixed with formalin and then embedded in paraffin. Samples were sectioned at a thickness of 4 μm and stained with hematoxylin and eosin (HE) for histopathologic analysis by light microscopy. An experienced pathologist evaluated the tissue sections and the severity of liver damage was graded using Suzuki score. The score consisted of three components (sinusoidal congestion, hepatocyte necrosis, and ballooning degeneration), which were graded from 0 to 4, as described by Suzuki et al. Followed by dewaxing hydration, antigen retrieval, staining, dehydration, mounting, and scanning, Image J software was used to analyze immunohistochemical staining images. IHC-anti-Foxp3 antibody was purchased from Abcam.

### Statistical Analysis

All data were presented as the mean ± SD for at least three independent experiments. Statistical analysis was performed by the two-sided Student's *t* test, ANOVA, or Kruskal–Wallis test when it does not conform to the normal distribution using GraphPad Prism7.0 software. Probability (*p*) values ≤ 0.05 were considered statistically significant. ^*^p < 0.05; ^**^p < 0.01; ^***^p < 0.001.

## Results

### Liver Microenvironment Acidification and Dysfunction Were Induced During HIRI in Humans and Mice

**Figure 1:**
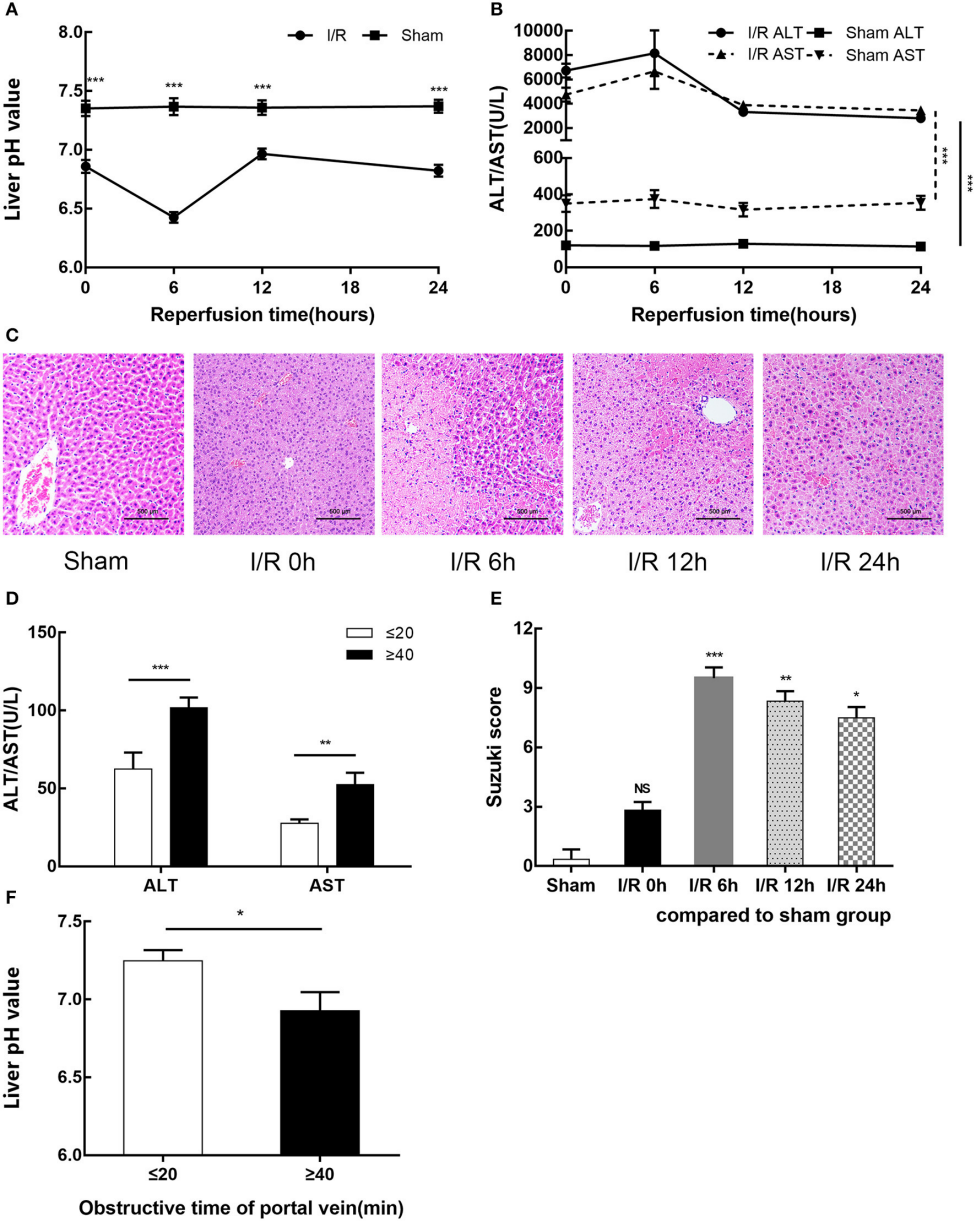
HIRI causes microenvironment acidification and dysfunction of the liver in humans and mice. **(A)** pH value of injured liver lobe by time point after reperfusion in mice. **(B)** Serum alanine aminotransferase (ALT) and aspartate transaminase (AST) levels in sham and I/R mouse groups. **(C)** Representative images of mouse liver injury [hematoxylin and eosin (HE) staining, 200×] by light microscopy. **(D)** Serum ALT and AST levels of 20 patients undergoing partial hepatectomy surgery by hepatic portal occlusion time after operation. **(E)** Suzuki scores evaluating liver biopsies by group, as performed by an experienced pathologist. **(F)** pH value of excised liver tissue of 20 patients undergoing partial hepatectomy surgery by hepatic portal occlusion time. Data are presented as the means ± SD from three independent experiments. *p < 0.05; **p < 0.01; ***p < 0.001.

We established a mouse model of partial hepatic ischemia/ reperfusion and collected injured liver lobe specimens and blood at 0, 6, 12, and 24 h after reperfusion. To evaluate the extent of liver damage, we measured the pH value of the ischemic liver lobe, detected increased serum ALT and AST, and performed HE staining of liver tissue. The I/R group exhibited an injured liver with a significantly lower pH after ischemia than the sham group; after 6 h of reperfusion, this pH reached a minimum and then gradually recovered (*p* < 0.001) (Figure 1A). Serum ALT and AST increased in the I/R group within 24 h, reached a peak at 6 h (8155.67 ± 1875.02 U/L and 6659.33 ± 1443.60 U/L, respectively) (*p* < 0.001), and then gradually recovered (Figure 1B). The pathological results of HE staining in the I/R 6-h group of mouse livers as evaluated by Suzuki score were consistent with the pH results: the most serious injury and most abnormal liver pH appeared at 6 h after reperfusion (Figures 1C,E). We also tested the relationship between pH and liver function in the clinic among 20 patients who underwent partial hepatectomy surgery. These patients were grouped by hepatic portal occlusion within 20 min or more than 40 min. We measured the pH of the resected liver tissue immediately after the liver tissue was excised. We found that the shorter the hepatic portal occlusion time, the less the pH dropped (Figure 1F). After the operation, we performed serological examination on both groups of patients. In accordance with the results in mice, both ALT and AST in the two groups were increased and the liver was found to recover faster with shorter hepatic portal occlusion time (Figure 1D). However, for ethical reasons, the pH value of normal human livers could not be determined.

### Acidic Microenvironment Decreases the Generation of CD4^+^CD25^+^Foxp3^+^iTregs

**Figure 2:**
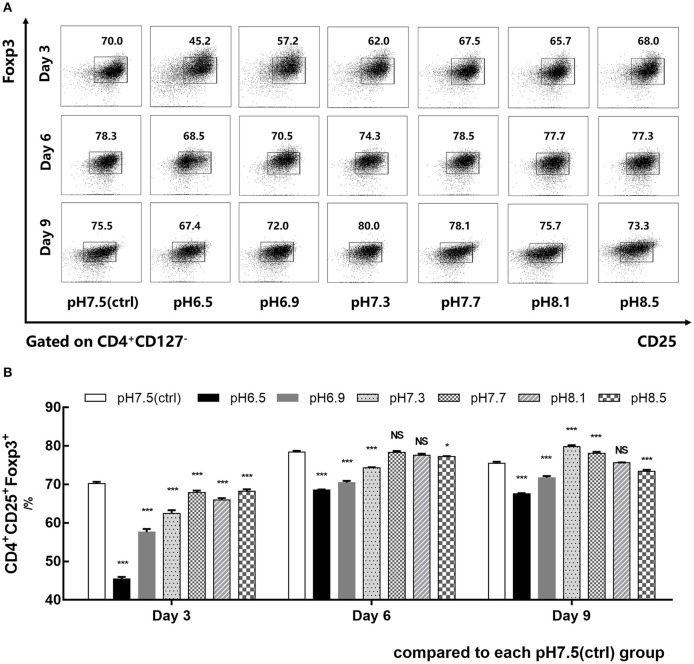
Acidic microenvironment decreases CD4^+^CD25^+^Foxp3^+^ iTreg induction by proapoptosis. **(A)** Induction efficiency of CD4^+^CD45RA^+^ naïve T cells cultured with IL-2 and TGF-β for 9 days at the indicated pH values. **(B)** Statistical analysis of induction efficiency. Data are presented as the means ± SD from three independent experiments. NS, not significant. *p < 0.05; ***p < 0.001.

CD4^+^CD45RA^+^ naïve T cells isolated from human PBMCs were co-cultured with IL-2, TGF-β, and anti-CD3/CD28 mAb-coated Dynabeads for 9 days in media of different pH values (6.5–8.5). Foxp3 expression of iTregs was detected by flow cytometry on days 3, 6, and 9. Data showed that the induction efficiency of iTregs in the pH 6.5 group was the lowest and its Foxp3 expression was significantly lower than the pH 7.5 group, especially on day 3. In addition, its induction rate was only 45.48% ± 0.50%, which was about 25% lower than that of the pH 7.5 group (*p* < 0.001) (Figures 2A,B). Therefore, the pH 7.5 and pH 6.5 groups were selected for further study.

**Figure 3:**
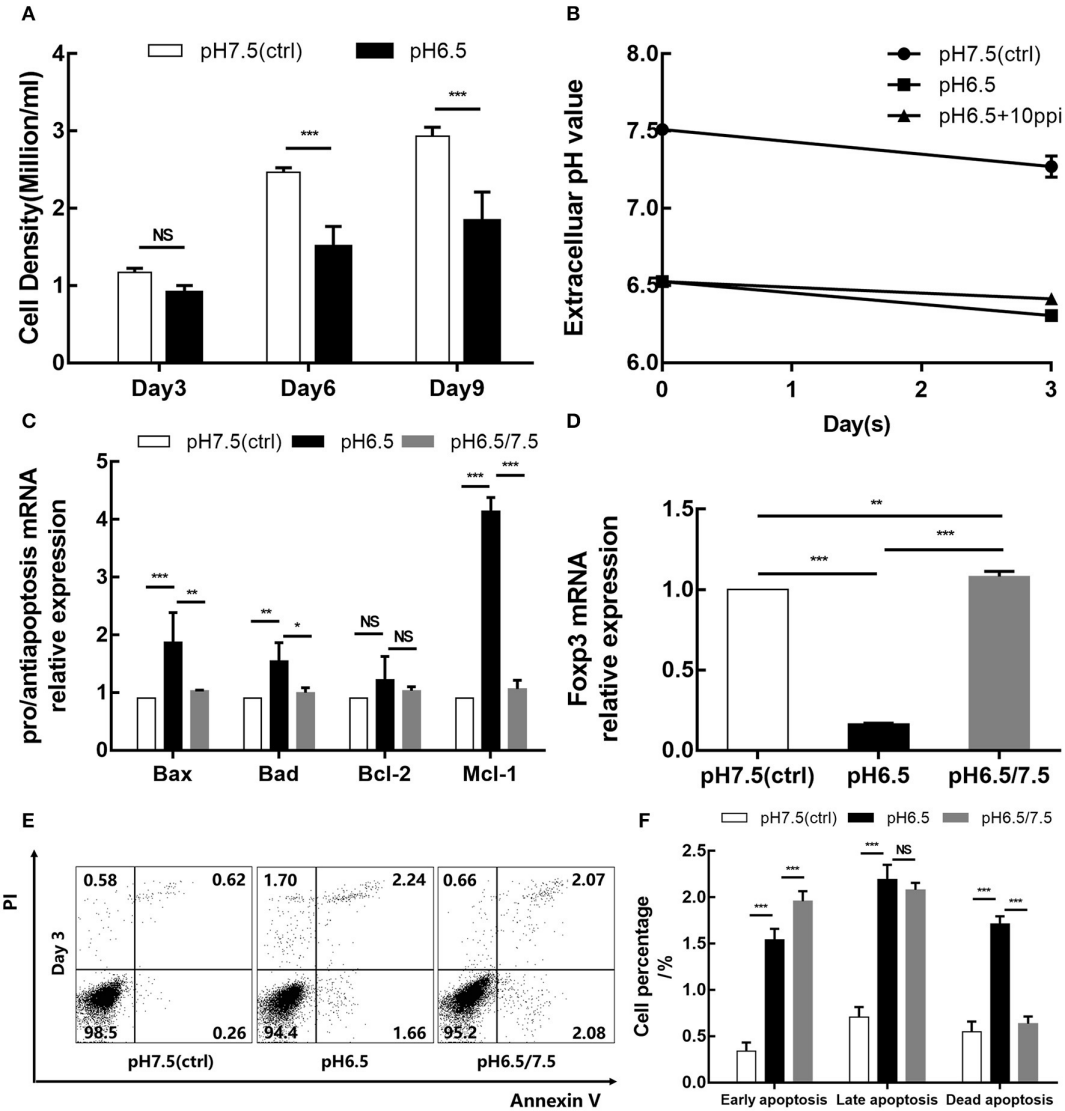
Acidic microenvironment downregulates the iTreg proliferation and Foxp3 expression and promotes apoptosis of iTregs. **(A)** Cell density of the pH 6.5 and pH 7.5 groups by time point. **(B)** Changes in extracellular pH during cell culture. **(C)** Expression of pro-apoptotic genes (*Bax* and *Bad*) and anti-apoptosis genes (*Bcl-2* and *Mcl-1*) measured by qRT-PCR at the transcriptional level after 3 days of culture. **(D)** mRNA expression of Foxp3 in iTregs measured by qRT-PCR at the indicated pH values after 3 days of culture. **(E)** Cell apoptosis after Annexin V/PI staining at the indicated pH values. **(F)** Statistical analysis of cell apoptosis by pH group. Data are presented as the means ± SD from three independent experiments. NS, not significant. *p < 0.05; **p < 0.01; ***p < 0.001.

Cell counts were performed on days 3, 6, and 9. The results showed that the density of the pH 6.5 group was lower than that of the pH 7.5 group, especially on days 6 and 9 (*p* < 0.001; *p* < 0.001) (Figure 3A). It is widely believed that an acidic microenvironment is not suitable for cell culture. Thus, to consider whether an acidic microenvironment induces the apoptosis of Tregs, we examined the cell apoptosis of the two groups on day 3. Additionally, changes in extracellular pH were also detected during cell culture (Figure 3B). After 3 days of culture, Annexin V–PI staining showed that the pH 6.5 group had significantly greater percentages of early and late apoptotic cells than the pH 7.5 group (*p* < 0.001), which may be one of reasons for cell count decline (Figures 3E,F). Meanwhile, transcript levels of pro-apoptotic and anti-apoptotic genes and Foxp3 in the two groups were detected. The expression of pro-apoptotic genes *Bax* and *Bad* was elevated in the pH 6.5 group; the expression of anti-apoptosis gene *Mcl-1* was more than four times that of the pH 7.5 group (*p* < 0.001) (Figure 3C). Furthermore, mRNA expression of Foxp3 was significantly lower in the pH 6.5 group than in the pH 7.5 group (*p* < 0.001) (Figure 3D).

### Reversal of Acidic Microenvironment Restores Foxp3 Expression and Regulatory Function of iTregs

**Figure 4:**
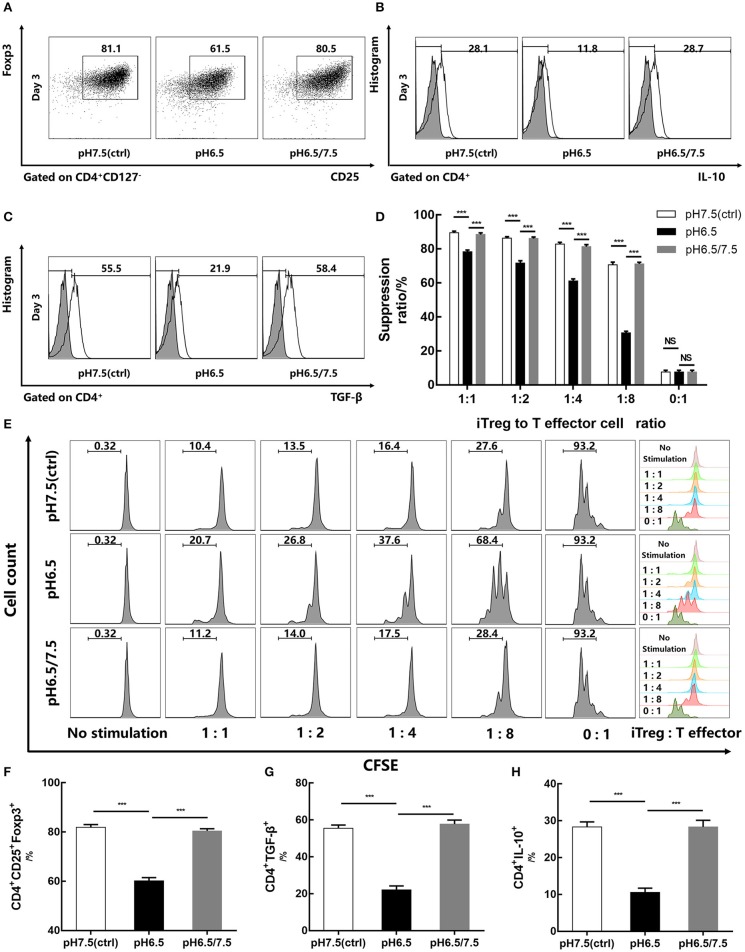
Reversal of acidic microenvironment restores Foxp3 expression and iTreg function. **(A)** The proportion of CD4^+^CD25^+^Foxp3^+^ iTregs cultured with IL-2 and TGF-β for 3 days. **(B,C)** The proportion of CD4^+^IL-10^+^ and CD4^+^ TGF-β^+^ T cells cultured with IL-2 and TGF-β for 3 days and stimulated with phorbol 12-myristate 13-acetate, brefeldin A, and ionomycin for 6 h before the assay. **(D)** Statistical analysis of the suppression assay *in vitro*. **(E)** iTregs induced in media of varying pH values for 3 days were co-cultured with carboxyfluorescein succinimidyl ester-stained human naïve CD4^+^ T cells (responders) at the indicated ratio. After 72 h of activation with anti-CD3/CD28-conjugated beads, responder cell proliferation was assessed by flow cytometry. **(F–H)** Statistical analysis of related results of flow cytometry. Data are presented as the means ± SD from three independent experiments. ***p < 0.001.

To investigate the effect of an acidic microenvironment on the function of iTregs induced from naïve T cells, changes in cytokines of TGF-β and IL-10 secreted by iTregs from different pH media were assessed by flow cytometry after 3 days of culture. Interestingly, TGF-β and IL-10 production, as well as Foxp3 expression, was restored when cells first cultured in a preconditioned medium (pH 6.5) were then returned to approximate physiological pH (7.5) for 24 h before the assay, indicating that the reversal of the acidic microenvironment helps restore functions of iTregs at least under these experimental settings (*p* < 0.001) (Figures 4A–C,F–H). This recovery phenomenon was also manifested in the pro-apoptotic genes *Bax* (*p* < 0.001) and *Bad* (*p* < 0.05) and the anti-apoptotic genes *Bcl-2* and *Mcl-1* (*p* < 0.001), as well as the mRNA expression of Foxp3 (*p* < 0.001) (Figures 3C,D). However, early apoptosis and late apoptosis cell levels of the pH 6.5/7.5 group did not decline, and even the early apoptosis level was higher than that of the pH 6.5 group (*p* < 0.001) (Figures 3E,F). We presume that this abnormal trend was caused by mechanical damage to the cells during centrifugation when the medium was replaced.

*In vitro* suppression assays were performed on pH 6.5, pH 7.5, and pH 6.5/7.5 groups of iTregs to evaluate their inhibitory function. From the results of the suppression assays, when the ratio of iTregs to effector T cells was 1:8, the inhibition function of the pH 6.5 group was significantly reduced, and the inhibition rate was only half of that of pH 7.5 group (70.67 ± 1.53% and 30.60 ± 0.91%, respectively) (*p* < 0.001) (Figures 4D,E).

### PI3K/Akt/mTOR Signaling Pathway Is Involved in the Regulation of iTregs by an Acidic Microenvironment

**Figure 5:**
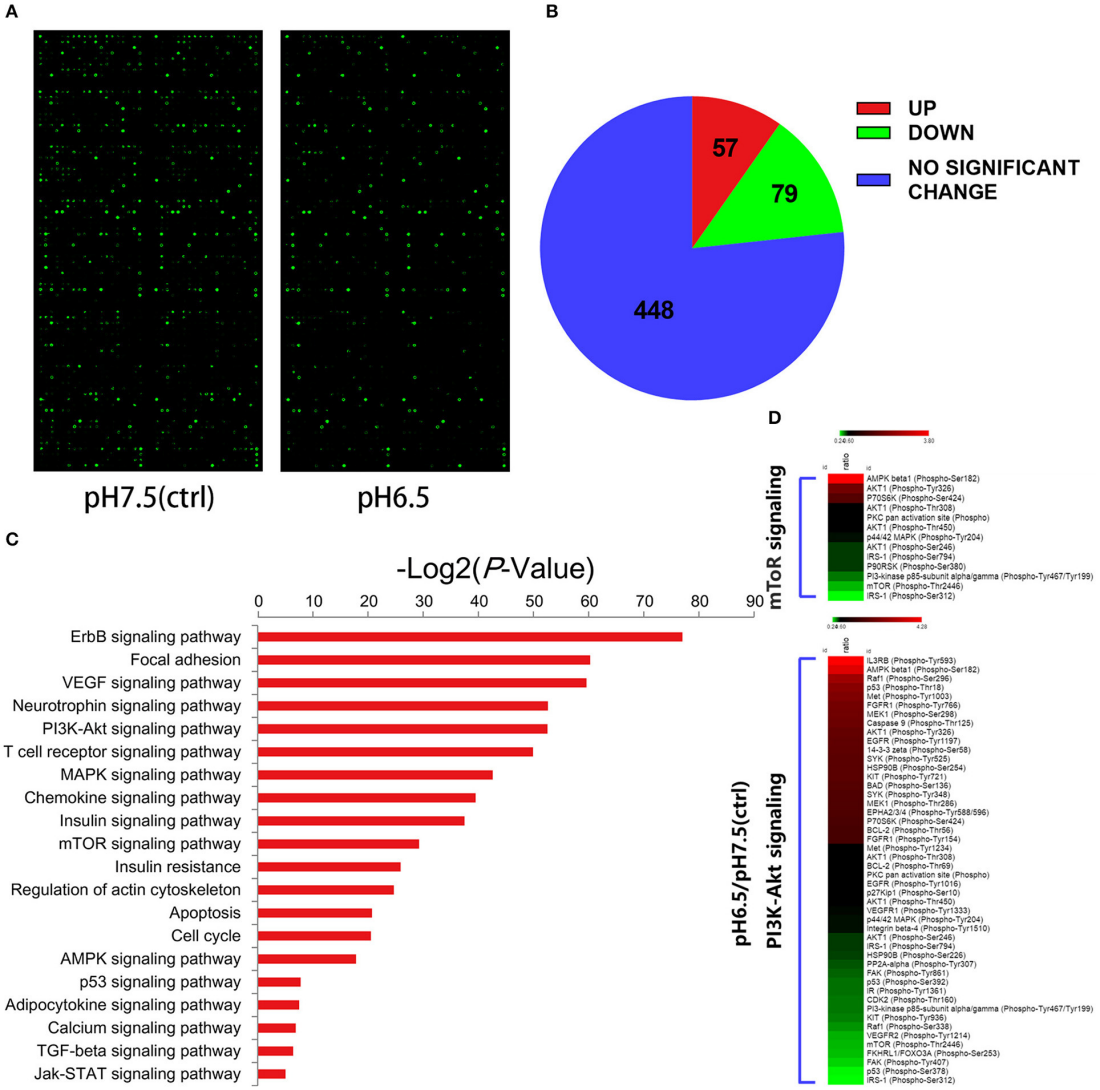
Differential expression of phospho-specific protein between cells in the pH 7.5 and pH 6.5 groups. **(A)** Original images of phosphorylated protein chips by SureScan Dx Microarray Scanner from two groups. **(B)** Phosphorylation sites (582) of all (432) screened proteins based on a phospho-specific protein microarray analysis. **(C)** KEGG enrichment analysis of proteins in the phospho-specific protein microarray according to cellular component, biological process, and molecular function. **(D)** Heat map representing the fold change in the expression of different phosphorylated sites of proteins in the PI3K/Akt/mTOR signaling pathway (fold change Phos/Unphos ≥ 1.6).

To better understand the effect of an acidic microenvironment on the signaling pathways of iTreg induction, we used a phospho-specific protein microarray containing 1,318 phosphoprotein antibodies to characterize the difference in phosphorylation patterns between pH 7.5 and pH 6.5 cells (Figure 5A). The pH 6.5 group exhibited 136 phosphorylation sites that significantly changed (57 phosphorylation sites significantly increased and 79 phosphorylation sites decreased compared to the pH 7.5 group) (fold change pH 6.5 vs. pH 7.5 ≥ 1.6) (Figure 5B). We performed a Kyoto Encyclopedia of Genes and Genomics analysis to determine enrichment pathways in iTregs induced by acidic pH (Figure 5C). The results of this analysis indicated that the PI3K/Akt/mTOR pathway was activated in iTregs of the pH 6.5 group. Previous studies have shown that the activation of the PI3K/Akt/mTOR signaling pathway leads to a decline in Foxp3 expression (18). Phosphorylation of the Ser2448 site is usually recognized as a marker for mTOR activation (19). However, the microarray results showed that no significant difference in phosphorylation was found between the two groups at the mTOR Ser2448 and Ser2481 sites. By contrast, phosphorylation at the Thr2446 site in the pH 7.5 group was more obvious than that in the pH 6.5 group (Figure 5D).

**Figure 6:**
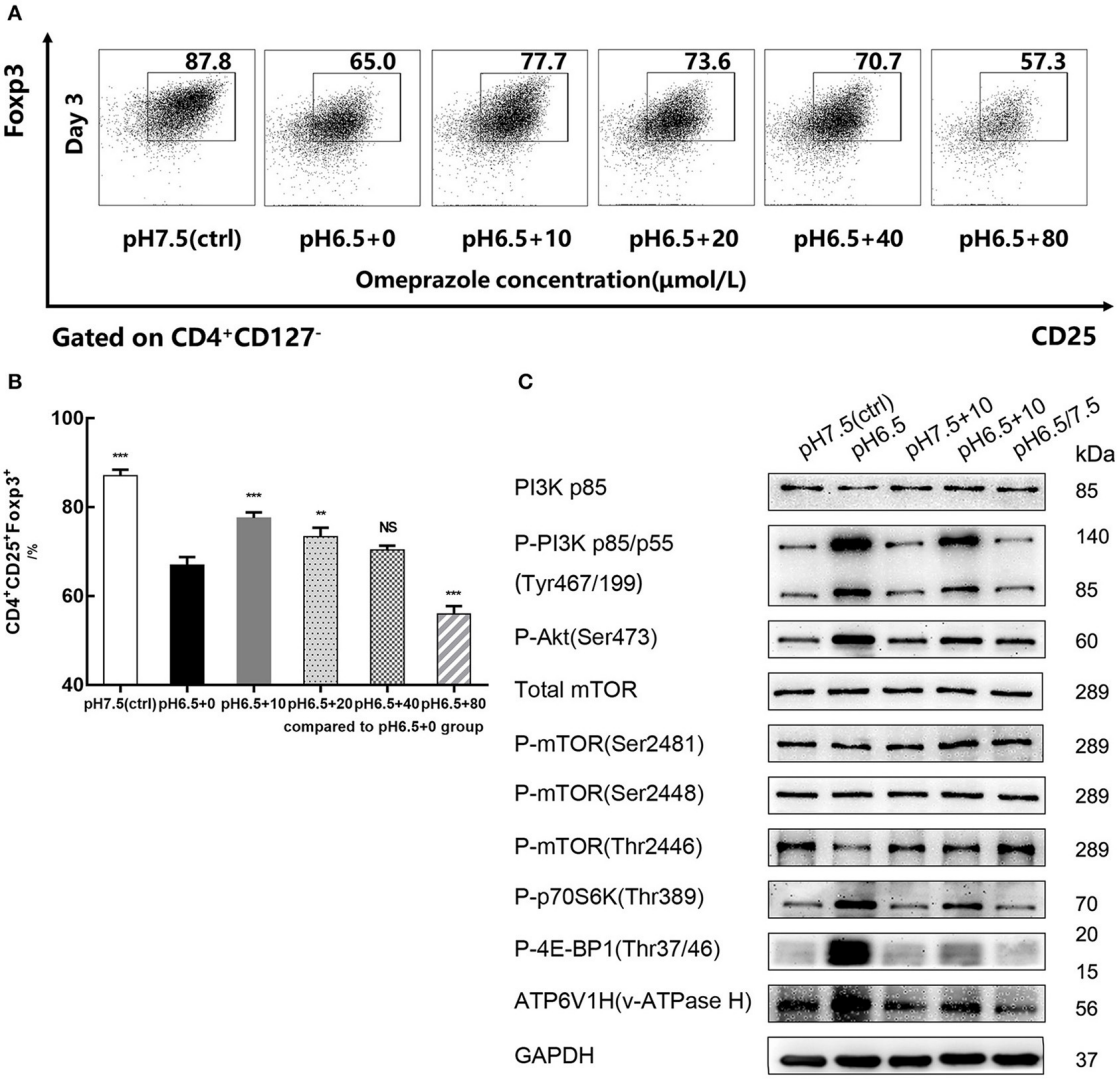
Omeprazole reverses the decreased iTreg differentiation caused by acidic pH. **(A)** Induction efficiency of CD4^+^CD45RA^+^ naïve T cells cultured with varying concentrations of omeprazole for 3 days at the indicated pH values. **(B)** Statistical analysis of induction efficiency of different groups. **(C)** Cells of different groups were harvested and subjected to SDS-PAGE and Western blot with the indicated antibodies. Data are presented as the means ± SD from three independent experiments. NS, not significant. **p < 0.01; ***p < 0.001.

We then confirmed our microarray results using Western blot analysis (Figure 6C). The results of the Western blot confirmed the results of three phosphorylation sites (Ser2448, Ser2481, Thr2446) of mTOR and total mTOR. To further validate this pathway, we performed Western blot analysis on both upstream and downstream signaling proteins of mTOR. Compared with the pH 7.5 group, the expression of P-PI3K p85/p55 and P-Akt phosphorylation proteins was increased in the pH 6.5 group, as was the expression of P-p70S6K and P-4E-BP1. By restoring the medium pH of the pH 6.5 group to approximate physiological pH (7.5), the results of the Western blot in the pH 6.5/7.5 group were similar to those in the pH 7.5 group. This similarity indicates that the reversal of the acidic microenvironment can restore the phenotype and function of iTregs, as supported by evidence in the signaling pathway (Figure 6C).

### Omeprazole Reverses Decrease of iTreg Differentiation Caused by an Acidic Microenvironment

After naïve T cells were treated for 72 h with varying concentrations of omeprazole in the medium of pH 6.5, the induction efficiency of CD4^+^CD25^+^Foxp3^+^iTregs decreased in a dose-dependent manner compared with that of the control group (pH 6.5 + 0 μmol/L) (*p* < 0.05). When the concentration of omeprazole reached 80 μmol/L, the ratio of iTregs was only 56.0 ± 1.76%, which probably resulted from the toxicity of the omeprazole solvent dimethyl sulfoxide (Figure 6A). When the concentration of omeprazole was 10 μmol/L, the induction efficiency of iTregs was higher than that of the control group (pH 6.5 + 0 μmol/L) (*p* < 0.001) but lower than that of the pH 7.5 group (87.1% ± 1.39%) (*p* < 0.001) (Figure 6B). Subsequently, the pH 7.5, pH 6.5, pH 7.5 + 10 μmol/L, pH 6.5 + 10 μmol/L, and pH 6.5/7.5 groups were prepared for Western blot analysis. Data suggested that omeprazole treatment partially inhibited the PI3K/Akt/mTOR signaling pathway, characterized by the decreased expression of P-PI3K (p85/p55), P-Akt, P-p70S6K, and P-4E-BP1 and increased P-mTOR (Thr2446) expression (Figure 6C). However, the pH 7.5+10 μmol/L group showed no significant differences compared to the physiologic pH 7.5 group.

Because omeprazole are prodrugs activated by protonation at low pH, their effect on lymphocytes should be selectively triggered by an acidic environment (20). In addition, V-ATPases (membrane proton pumps) were also detected by Western blot. The results showed that the pH 6.5 group had greater V-ATPase expression than the pH 7.5 group, and this expression significantly decreased after treatment with omeprazole.

### Buffering of the Acidic Microenvironment Increases Treg Infiltration and Alleviates Liver Injury in a HIRI Mouse Model

**Figure 7:**
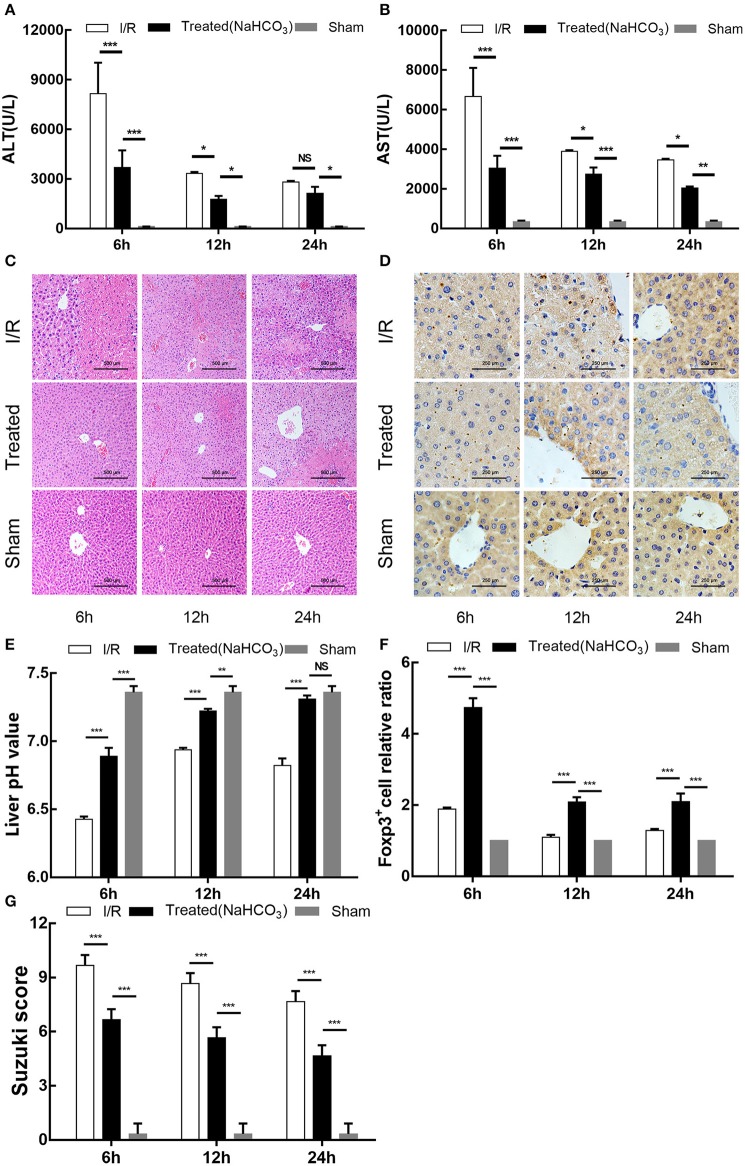
Buffering of acidic microenvironment increases Treg infiltration and alleviates liver injury in HIRI mouse model. **(A,B)** Serum ALT and aspartate transaminase (AST) levels in the I/R, NaHCO_3_ treatment, and sham mouse groups by time points. **(C)** Representative light microscopy images of mouse liver injury (HE staining, 200×) in the I/R, NaHCO_3_ treatment, and sham groups by time point. **(D)** Representative light microscopy images of mice liver immunohistochemical staining in the I/R, NaHCO_3_ treatment, and sham groups (brown: positive cells, anti-Foxp3, 400×) by time point. **(E)** pH values of injured liver lobe after reperfusion in I/R, NaHCO_3_ treatment, and sham mouse groups by time point. **(F)** Statistical analysis of Foxp3^+^ cell relative ratio of I/R, NaHCO_3_ treatment, and sham mouse groups in immunohistochemical staining. **(G)** Suzuki scores evaluating liver biopsies from different groups, as performed by an experienced pathologist. Data are presented as the means ± SD from three independent experiments. NS, not significant. *p < 0.05; **p < 0.01; ***p < 0.001.

Similar to the previously established partial hepatic ischemia/reperfusion mice model, injured liver lobe tissue and blood of NaHCO_3_ treatment mice group was collected at 6 h, 12 h, and 24 h after reperfusion. In addition to measuring the pH of damaged liver lobe tissue and detecting serum ALT and AST, we also performed HE and immunohistochemical staining (Foxp3) on the liver tissue to assess the degree of damage and the extent of Treg infiltration. Consistent with the previous experimental results, the pH of injured liver lobe tissue in the I/R group reached its minimum at 6 h after reperfusion and then increased at 24 h after reperfusion (*p* < 0.001); however, the level of pH value was lower than NaHCO_3_ treatment mice group across the study duration (*p* < 0.001) (Figure 7E). The degree of liver damage of the treatment group was improved compared with the I/R group (*p* < 0.05) (Figures 7A–C). The immunohistochemical results of Foxp3 in the mouse liver showed that Treg infiltration was significantly increased in the treated group (*p* < 0.001), reaching its maximum at 6 h after reperfusion and then gradually decreasing (Figures 7D,F). Our experimental results indicate that the reversal of the acidic microenvironment in the HIRI mouse model can increase Treg infiltration and alleviate liver injury.

### Reversal of Acidic Microenvironment Attenuating HIRI in Mice Is Associated With Tregs

**Figure 8:**
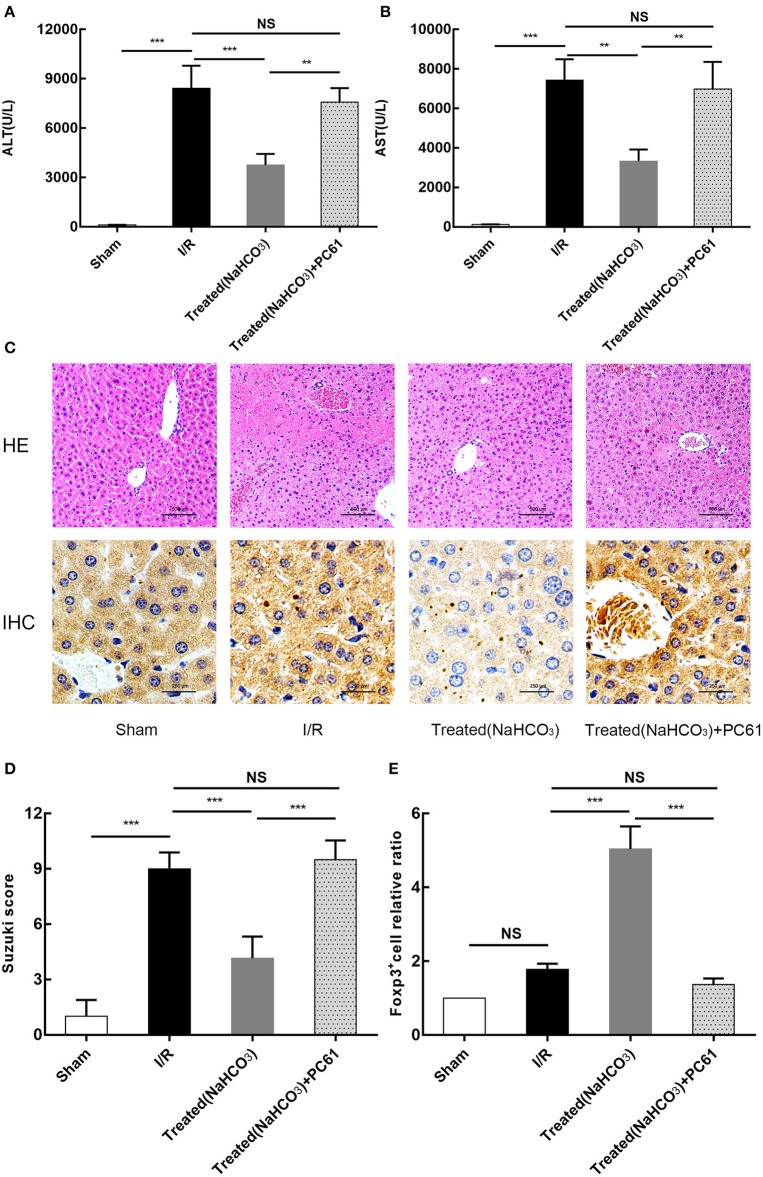
Reversal of acidic microenvironment attenuates HIRI in mice via Treg function. **(A,B)** Serum ALT and aspartate transaminase (AST) levels of mice in sham, I/R, NaHCO_3_ treatment, and NaHCO_3_ + PC61 treatment groups after reperfusion for 6 h. **(C)** Representative images of mouse liver injury (HE staining, 200×; immunohistochemical staining, 400×) in sham, I/R, NaHCO_3_ treatment, and NaHCO_3_ + PC61 treatment groups by light microscopy after reperfusion for 6 h. **(D)** Suzuki scores evaluating liver biopsies from different groups, as performed by an experienced pathologist. **(E)** Statistical analysis of Foxp3^+^ cells relative ratio of sham, I/R, NaHCO_3_ treatment, and NaHCO_3_ + PC61 treatment groups in immunohistochemical staining. Data are presented as the means ± SD from three independent experiments. NS, not significant. **p < 0.01; ***p < 0.001.

All above studies showed that the reversal of the acidic microenvironment can alleviate HIRI in mice. To further clarify the role of Tregs in buffering of the acidic microenvironment to reduce HIRI, PC61 (CD25 antibody) was used in mice. PC61 can effectively inhibit the function of Tregs. From the results of ALT and AST, as well as HE and immunohistochemical staining (Foxp3) (Figure 8), this reversal effect was abolished in mice with PC61 treatment. This finding suggests that Tregs play a role in the reversal of the acidic microenvironment to alleviate HIRI.

## Discussion

It is evident from the above review that the pH of the extracellular milieu has a direct influence on immune cells function. To investigate the effects of different pH values on iTregs induction, we performed a series of experiments *in vitro* and vivo and found that an acidic microenvironment in HIRI can inhibit the generation and function of CD4^+^CD25^+^Foxp3^+^ iTregs via the PI3K/Akt/mTOR signaling pathway. By contrast, the reversal of the acidic microenvironment restored Foxp3 expression and iTreg function.

During partial hepatectomy or liver transplantation, HIRI can cause acute liver injury, affecting the recovery of distant organs after surgery and even leading to patient death (21). In recent years, studies have found that T cells are the primary mediators of HIRI (22). CD4^+^ T cells are an important regulator of hepatic neutrophil recruitment during HIRI via the release of IL-17 (23). IL-17 is also released by RORγt^+^CD3^+^ T cells and is involved in HIRI by recruiting neutrophil and Kupffer cell activation (24). In addition, the reduction or elimination of CD4^+^ natural killer T cells can effectively inhibit the aggregation and activation of natural killer T cells and neutrophils, thereby reducing HIRI (25).

Tregs are T cells with immunosuppressive function and are extremely important for maintaining immune system homeostasis in the body. Tregs are closely associated with a variety of diseases such as malignant tumors (26), autoimmune diseases (27), infectious diseases (28), and transplant rejection (29). In our previous studies, pretreatment with iTregs ameliorated HIRI and decreased IFN-γ and IL-17 in the liver after reperfusion. Recently, Zheng et al. found that exosomes produced by bone marrow–derived dendritic cells alleviate HIRI via regulating the balance between Tregs and Th17 cells (30).

The importance of acid–base homeostasis in maintaining normal cellular responses and physiological activity has been recognized for a long time (31). When the extracellular pH decreases, many cellular responses are diminished, including cytoplasmic- and membrane-associated enzyme activities, ion transport activity, protein and DNA synthesis, and cAMP and calcium levels (32). Studies that have been conducted tend to focus on the influence of pH on lymphocytes in the tumor microenvironment. Interestingly, extracellular acidosis can lead to stimulation as well as suppression of immune cell activity. For example, an acidic microenvironment stimulates the expression of nitric oxide synthase in macrophages (33) and the activity of neutrophils (34) but suppresses cytotoxic lymphocytes and natural killer cells (35). Thus, the effect of the extracellular acidic microenvironment on lymphocytes may be different. Similar to the research of Calcinotto et al., whose study showed that extracellular microenvironment acidification (pH, 6.0–6.5) has a negative effect on the function of human and mammalian tumor-infiltrating T cells, resulting in a decrease in its anti-tumor ability (36), our data also demonstrated that acidic microenvironment in HIRI downregulates the iTreg function and Foxp3 expression and promotes apoptosis of iTregs.

Previous evidence has suggested that an acidic microenvironment activates the PI3K/AkT signaling pathway and significantly enhances Akt phosphorylation (37). Most cell growth and metabolism are associated with the PI3K/Akt/mTOR signaling pathway (38). In particular, the mTOR pathway plays a central role in the development of various diseases. In the current study, we found that acidic microenvironment inhibits the phosphorylation of the mTOR Thr2446 site during induction. Although basic science research on this Thr2446 site is rare, it is generally considered to be a phosphorylation site associated with negative regulation of mTOR activity, acting as an opposing role against the Ser2448 site. Both phosphorylation sites may act as a switch to control the positive and negative signals regulating protein translation (39). However, we have no way to interfere with the phosphorylation of the mTOR Thr2446 site due to the lack of specific phosphorylation inhibitors. Fortunately, by restoring the pH of the medium, we observed that the mTOR Thr2446 site was re-phosphorylated, which also indicates that an acidic microenvironment can specifically inhibit phosphorylation at this site. Therefore, we believe that the PI3K/Akt/mTOR signaling pathway is involved in the regulation of iTregs by an acidic microenvironment and that the mTOR threonine 2,446 phosphorylation site may be a novel phosphorylation site for mTOR activation, which is associated with Foxp3 induction.

To our knowledge, this study is the first to confirm the presence of V-ATPases in iTregs. V-ATPases are primarily located on the membrane of internal acidic vesicles and plasma membranes and play an important role in regulating the transmembrane pH gradient (40–42). Our study demonstrated that iTregs express more V-ATPases under an acidic microenvironment and the proton pump inhibitor omeprazole can reduce this expression. In addition, omeprazole can inhibit the activation of the PI3K/Akt/mTOR signaling pathway under acidic conditions, and it appears that the inhibition of the PI3K/Akt/mTOR signaling pathway is mediated through V-ATPases. According to our cell culture results, omeprazole can also improve the decreased iTreg differentiation caused by the acidic microenvironment, indicating its potential clinical use in the treatment of HIRI.

Although we found that the proton pump inhibitor omeprazole can increase the induction efficiency of iTreg in acidic microenvironment and inhibit the PI3K/Akt/mTOR signaling pathway and V-ATPase expression, further research is needed. In addition, we have not attempted to directly apply omeprazole to HIRI mice model to observe whether it can change the liver acidic microenvironment and alleviate HIRI, which is also needed to be further studied.

Finally, we conclude that an acidic microenvironment is a key regulator in HIRI, involved in modulating the generation and function of Tregs. An acidic microenvironment can inhibit the generation and function of CD4^+^CD25^+^Foxp3^+^ iTregs via activating the PI3K/Akt/mTOR signaling pathway and reversing the acidic microenvironment to restore Foxp3 expression and iTreg function. In addition, this decreased iTregs differentiation caused by the acidic microenvironment can be partially improved by the proton pump inhibitor omeprazole. Furthermore, buffering the acidic microenvironment to attenuate HIRI in mice has an inseparable relationship with Tregs.

## Data Availability Statement

The raw data supporting the conclusions of this article will be made available by the authors, without undue reservation, to any qualified researcher.

## Ethics Statement

The studies involving human participants were reviewed and approved by Ethics Committee of the First Affiliated Hospital of Nanjing Medical University. The patients/participants provided their written informed consent to participate in this study. The animal study was reviewed and approved by Institutional Animal Care and Research Advisory Committee of Nanjing Medical University.

## Author Contributions

XG, RZ, and JG designed the experiments, performed the experiments, analyzed the data, and wrote the manuscript. XG, ZJ, XW, QW, HP, JQ, and JZ performed the experiments and interpreted the data. LL and FC designed the overall concept, analyzed the data, and wrote the manuscript.

### Conflict of Interest

The authors declare that the research was conducted in the absence of any commercial or financial relationships that could be construed as a potential conflict of interest.

## References

[1] Shuh M Bohorquez H Loss GE Jr Cohen AJ. Tumor necrosis factor-alpha: life and death of hepatocytes during liver ischemia/reperfusion injury. Ochsner J. (2013) 13:119–30. 23531747 PMC3603175

[2] Li J Li RJ Lv GY Liu HQ. The mechanisms and strategies to protect from hepatic ischemia-reperfusion injury. Eur Rev Med Pharmacol Sci. (2015) 19:2036–47. 26125267

[3] Guan LY Fu PY Li PD Li ZN Liu HY Xin MG. Mechanisms of hepatic ischemia-reperfusion injury and protective effects of nitric oxide. World J Gastrointest Surg. (2014) 6:122–8. 10.4240/wjgs.v6.i7.122 25068009 PMC4110529

[4] He Z Ma C Yu T Song J Leng J Gu X. Activation mechanisms and multifaceted effects of mast cells in ischemia reperfusion injury. Exp Cell Res. (2019) 376:227–35. 10.1016/j.yexcr.2019.01.022 30716302

[5] Datta G Fuller BJ Davidson BR. Molecular mechanisms of liver ischemia reperfusion injury: insights from transgenic knockout models. World J Gastroenterol. (2013) 19:1683–98. 10.3748/wjg.v19.i11.1683 23555157 PMC3607745

[6] Martin M Mory C Prescher A Wittekind C Fiedler M Uhlmann D. Protective effects of early CD4(+) T cell reduction in hepatic ischemia/reperfusion injury. J Gastrointest Surg. (2010) 14:511–9. 10.1007/s11605-009-1104-3 19937475

[7] Xie K Liu L Chen J Liu F. Exosomal miR-1246 derived from human umbilical cord blood mesenchymal stem cells attenuates hepatic ischemia reperfusion injury by modulating T helper 17/regulatory T balance. IUBMB Life. (2019) 71:2020–30. 10.1002/iub.2147 31433911

[8] Sharabi A Tsokos MG Ding Y Malek TR Klatzmann D Tsokos GC. Regulatory T cells in the treatment of disease. Nat Rev Drug Discov. (2018) 17:823–44. 10.1038/nrd.2018.148 30310234

[9] Lu L Barbi J Pan F. The regulation of immune tolerance by FOXP3. Nat Rev Immunol. (2017) 17:703–17. 10.1038/nri.2017.75 28757603 PMC5793224

[10] Lu L Li G Rao J Pu L Yu Y Wang X. *in vitro* induced CD4(+)CD25(+)Foxp3(+) Tregs attenuate hepatic ischemia-reperfusion injury. Int Immunopharmacol. (2009) 9:549–52. 10.1016/j.intimp.2009.01.020 19539564

[11] Kraus M Wolf B Implications of acidic tumour microenvironment for neoplastic growth and cancer treatment: a computer analysis. Tumour Biol. (1996) 17:133–54. 10.1159/000217977 8638088

[12] Böhme I Bosserhoff AK. Acidic tumor microenvironment in human melanoma. Pigment Cell Melanoma Res. (2016) 29:508–23. 10.1111/pcmr.12495 27233233

[13] Sutoo S Maeda T Suzuki A Kato Y. Adaptation to chronic acidic extracellular pH elicits a sustained increase in lung cancer cell invasion and metastasis. Clin Exp Metastasis. (2019) 10.1007/s10585-019-09990-1. [Epub ahead of print]. 31489536 PMC7007909

[14] Inoue S Krieger I Sarnaik A Ravindranath Y Fracassa M Ottenbreit MJ. Inhibition of bone marrow stem cell growth *in vitro* by methylmalonic acid: a mechanism for pancytopenia in a patient with methylmalonic acidemia. Pedia1tr Res. (1981) 15:95–8. 10.1203/00006450-198102000-00001 7254944

[15] Church JA Koch R Shaw KN Nye CA Donnell GN. Immune functions in methylmalonicaciduria. J Inherit Metab Dis. (1984) 7:12–4. 10.1007/BF01805612 6429434

[16] Menkin V Diabetics and inflammation. Science. (1941) 93:456–64. 10.1126/science.93.2419.456 17820718

[17] Lentsch AB Yoshidome H Cheadle WG Miller FN Edwards MJ. Chemokine involvement in hepatic ischemia/reperfusion injury in mice: roles for macrophage inflammatory protein-2 and Kupffer cells. Hepatology. (1998) 27:507–12. 10.1002/hep.510270226 9462650

[18] Sauer S Bruno L Hertweck A Finlay D Leleu M Spivakov M. T cell receptor signaling controls Foxp3 expression via PI3K, Akt, and mTOR. Proc Natl Acad Sci USA. (2008) 105:7797–802. 10.1073/pnas.0800928105 18509048 PMC2409380

[19] Bolster DR Crozier SJ Kimball SR Jefferson LS. AMP-activated protein kinase suppresses protein synthesis in rat skeletal muscle through down-regulated mammalian target of rapamycin (mTOR) signaling. J Biol Chem. (2002) 277:23977–80. 10.1074/jbc.C200171200 11997383

[20] Mullin JM Gabello M Murray LJ Farrell CP Bellows J Wolov KR. Proton pump inhibitors: actions and reactions. Drug Discov Today. (2009) 14:647–60. 10.1016/j.drudis.2009.03.014 19443264

[21] Rao J Qian X Li G Pan X Zhang C Zhang F. ATF3-mediated NRF2/HO-1 signaling regulates TLR4 innate immune responses in mouse liver ischemia/reperfusion injury. Am J Transplant. (2015) 15:76–87. 10.1111/ajt.12954 25359217

[22] Rao J Cheng F Yang S Zhai Y Lu L. Ag-specific CD4 T cells promote innate immune responses in liver ischemia reperfusion injury. Cell Mol Immunol. (2019) 16:98–100. 10.1038/s41423-018-0051-x 29907880 PMC6318264

[23] Caldwell CC Okaya T Martignoni A Husted T Schuster R Lentsch AB. Divergent functions of CD4+ T lymphocytes in acute liver inflammation and injury after ischemia-reperfusion. Am J Physiol Gastrointest Liver Physiol. (2005) 289:G969–76. 10.1152/ajpgi.00223.2005 16002566

[24] Eggenhofer E Rovira J Sabet-Baktach M Groell A Scherer MN Dahlke MH. Unconventional RORγt+ T cells drive hepatic ischemia reperfusion injury. J Immunol. (2013) 191:480–7. 10.4049/jimmunol.1202975 23740948

[25] Zimmerman MA Martin A Yee J Schiller J Hong JC. Natural killer T cells in liver ischemia–reperfusion injury. J Clin Med. (2017) 6:41. 10.3390/jcm6040041 28368299 PMC5406773

[26] Wolf D Sopper S Pircher A Gastl G Wolf AM Treg(s) in cancer: friends or foe? J Cell Physiol. (2015) 230:2598–605. 10.1002/jcp.25016 25913194

[27] Fasching P Stradner M Graninger W Dejaco C Fessler J. Therapeutic potential of targeting the Th17/Treg axis in autoimmune disorders. Molecules. (2017) 22:134. 10.3390/molecules22010134 28098832 PMC6155880

[28] Attias M Al-Aubodah T Piccirillo CA. Mechanisms of human FoxP3+ Treg cell development and function in health and disease. Clin Exp Immunol. (2019) 197:36–51. 10.1111/cei.13290 30864147 PMC6591147

[29] Bézie S Anegon I Guillonneau C. Advances on CD8+ Treg cells and their potential in transplantation. Transplantation. (2018) 102:1467–78. 10.1097/TP.0000000000002258 29688996

[30] Zheng L Li Z Ling W Zhu D Feng Z Kong L. Exosomes derived from dendritic cells attenuate liver injury by modulating the balance of Treg and Th17 cells after ischemia reperfusion. Cell Physiol Biochem. (2018) 46:740–56. 10.1159/000488733 29621784

[31] Busa WB Nuccitelli R. Metabolic regulation via intracellular pH. Am J Physiol. (1984) 246:R409–38. 10.1152/ajpregu.1984.246.4.R409 6326601

[32] Lardner A. The effects of extracellular pH on immune function. J Leukoc Biol. (2001) 69:522–30. 11310837

[33] Bellocq A Suberville S Philippe C Bertrand F Perez J Fouqueray B. Low environmental pH is responsible for the induction of nitric-oxide synthase in macrophages. Evidence for involvement of nuclear factor-kappaB activation. J Biol Chem. (1998) 273:5086–92. 10.1074/jbc.273.9.5086 9478960

[34] Martínez D Vermeulen M Trevani A Ceballos A Sabatté J Gamberale R. Extracellular acidosis induces neutrophil activation by a mechanism dependent on activation of phosphatidylinositol 3-kinase/Akt and ERK pathways. J Immunol. (2006) 176:1163–71. 10.4049/jimmunol.176.2.1163 16394005

[35] Fischer K Hoffmann P Voelkl S Meidenbauer N Ammer J Edinger M. Inhibitory effect of tumor cell-derived lactic acid on human T cells. Blood. (2007) 109:3812–9. 10.1182/blood-2006-07-035972 17255361

[36] Calcinotto A Filipazzi P Grioni M Iero M De Milito A Ricupito A. Modulation of microenvironment acidity reverses anergy in human and murine tumor-infiltrating T lymphocytes. Cancer Res. (2012) 72:2746–56. 10.1158/0008-5472.CAN-11-1272 22593198

[37] Rafiee P Theriot ME Nelson VM Heidemann J Kanaa Y Horowitz SA. Human esophageal microvascular endothelial cells respond to acidic pH stress by PI3K/AKT and p38 MAPK-regulated induction of Hsp70 and Hsp27. Am J Physiol Cell Physiol. (2006) 291:C931–45. 10.1152/ajpcell.00474.2005 16790501

[38] Moore T Beltran L Carbajal S Strom S Traag J Hursting SD. Dietary energy balance modulates signaling through the Akt/mammalian target of rapamycin pathways in multiple epithelial tissues. Cancer Prev Res. (2008) 1:65–76. 10.1158/1940-6207.CAPR-08-0022 19138937

[39] Cheng SW Fryer LG Carling D Shepherd PR. Thr2446 is a novel mammalian target of rapamycin (mTOR) phosphorylation site regulated by nutrient status. J Biol Chem. (2004) 279:15719–22. 10.1074/jbc.C300534200 14970221

[40] Tarrado-Castellarnau M de Atauri P Cascante M. Oncogenic regulation of tumor metabolic reprogramming. Oncotarget. (2016) 7:62726–53. 10.18632/oncotarget.10911 28040803 PMC5308762

[41] Koltai T. Cancer: fundamentals behind pH targeting and the double-edged approach. Onco Targets Ther. (2016) 9:6343–60. 10.2147/OTT.S115438 27799782 PMC5074768

[42] Nishi T Forgac M. The vacuolar (H+)-ATPases–nature's most versatile proton pumps. Nat Rev Mol Cell Biol. (2002) 3:94–103. 10.1038/nrm729 11836511

